# Cross-talk between subchondral bone and articular cartilage in osteoarthritis

**DOI:** 10.1186/ar3714

**Published:** 2012-03-08

**Authors:** Steven R Goldring

**Affiliations:** 1 Hospital for Special Surgery, New York 10021, NY, USA

## 

The interest in the relationship between articular cartilage and the structural and functional properties of peri-articular bone relates to the intimate contact that exists between these tissues in diarthrodial joints. Much of the interest and continuing controversy regarding the relationship between these tissues dates back to the original suggestion by Radin and Rose that alterations in the mechanical properties of subchondral bone could adversely affect the functional state of chondrocytes and the integrity of the overlying cartilage. They hypothesized that the deterioration in articular cartilage associated with the osteoarthritic process was secondary to an initial increase in subchondral bone stiffness induced by repetitive mechanical loading. Numerous approaches have been used to establish that the changes in periarticular bone occur very early in the development of OA. Although chondrocytes also have the capacity to modulate their functional state in response to loading, the capacity of these cells to repair and modify their surrounding extracellular matrix is relatively limited in comparison to the adjacent subchondral bone. This differential adaptive capacity likely underlies the more rapid appearance of detectable skeletal changes in OA in comparison to the articular cartilage. Given the intimate mechanical and biological interaction between the articular cartilage and subchondral bone it is likely that alterations in the composition and/or structural organization of either tissue will modulate the properties and function of the other joint component. An additional factor that affects the interaction between these tissues is the gradual expansion of the zone of calcified cartilage and advancement of the tidemark that occurs with aging and progression of OA and contributes to overall thinning of the articular cartilage. The precise mechanisms involved in this process have not been definitively established and could include the release of pro-angiogenic factors from chondrocytes in the deep zones of the articular cartilage that have undergone hypertrophy and/or the influences of microcracks that have initiated focal remodeling in the calcified cartilage in an attempt to repair the microdamage. In addition, this process markedly increases the mechanical stresses in the deep zones of the cartilage matrix, which likely contributes to the acceleration in OA cartilage deterioration. In summary, there is the need for further studies to define the pathophysiological mechanisms involved in the interaction between subchondral bone and articular cartilage and for applying this information to the development of therapeutic interventions to improve the outcomes in patients with OA.

